# Exploring differential item functioning in the Western Ontario and McMaster Universities Osteoarthritis Index (WOMAC)

**DOI:** 10.1186/1471-2474-13-265

**Published:** 2012-12-29

**Authors:** Beth Pollard, Marie Johnston, Diane Dixon

**Affiliations:** 1 Aberdeen Health Psychology Group, University of Aberdeen, Aberdeen, UK; 2 School of Psychological Sciences and Health, University of Strathclyde, Glasgow, UK

**Keywords:** Osteoarthritis, WOMAC, Psychometrics, Item bias, Differential item functioning, Measurement equivalence

## Abstract

**Background:**

The Western Ontario and McMaster Universities Osteoarthritis Index (WOMAC) is a widely used patient reported outcome in osteoarthritis. An important, but frequently overlooked, aspect of validating health outcome measures is to establish if items exhibit differential item functioning (DIF). That is, if respondents have the same underlying level of an attribute, does the item give the same score in different subgroups or is it biased towards one subgroup or another. The aim of the study was to explore DIF in the Likert format WOMAC for the first time in a UK osteoarthritis population with respect to demographic, social, clinical and psychological factors.

**Methods:**

The sample comprised a community sample of 763 people with osteoarthritis who participated in the Somerset and Avon Survey of Health. The WOMAC was explored for DIF by gender, age, social deprivation, social class, employment status, distress, body mass index and clinical factors. Ordinal regression models were used to identify DIF items.

**Results:**

After adjusting for age, two items were identified for the physical functioning subscale as having DIF with age identified as the DIF factor for 2 items, gender for 1 item and body mass index for 1 item. For the WOMAC pain subscale, for people with hip osteoarthritis one item was identified with age-related DIF. The impact of the DIF items rarely had a significant effect on the conclusions of group comparisons.

**Conclusions:**

Overall, the WOMAC performed well with only a small number of DIF items identified. However, as DIF items were identified in for the WOMAC physical functioning subscale it would be advisable to analyse data taking into account the possible impact of the DIF items when weight, gender or especially age effects, are the focus of interest in UK-based osteoarthritis studies. Similarly for the WOMAC pain subscale in people with hip osteoarthritis it would be worthwhile to analyse data taking into account the possible impact of the DIF item when age comparisons are of primary interest.

## Background

Osteoarthritis (OA) is the most common cause of disability in the UK [1] and with an aging population ever more treatments and procedures are being carried out. The increase in the number of treatments and procedures combined with limited resources means it is even more important to use accurate measures of outcome. Without good measures we cannot identify those that benefit from treatments or, indeed, identify those that do not benefit from treatments, such as joint replacement, and for whom possibly other less invasive treatments may be more appropriate.

The Western Ontario and McMaster Universities Osteoarthritis Index (WOMAC [2]) is the most commonly used disease-specific measure of outcome used in OA [3]. The WOMAC was based on the objective of defining the dimensionality of pain and disability in osteoarthritis of the hip and/or knee. The WOMAC has 24 items with a total score and three subscales: pain, stiffness and physical function.

An important, but frequently overlooked aspect of establishing the validity of a measure, is to establish if items and measures work in the same way across subgroups of a population e.g. certain socio-economic groups or gender. Importantly, the method allows for the detection of item bias independent of true differences in limitation between the groups. That is, if respondents have the same underlying level of an attribute, does the measure give the same score in different populations or is it biased in some groups? For example, it has been shown that for the Centre of Epidemiology Scale of Depression (CES-D), women are more likely than men to endorse an item about having crying spells even though they have the same underlying level of depression [4]. Thus, this item exhibits bias with respect to gender and scores for women might be inflated compared to men. Hence, apparent group differences may be due to measurement bias rather than true differences. Although a measure may appear equivalent at the measure level, biases may still be present at the individual item level [5]. Thus, item level analyses are now seen as central to establishing measurement equivalence across subgroups of a population [6].

Methods of detecting item level bias have been developed in the area of education testing designed to avoid biases, such as, for different racial groups. These methods are beginning to be implemented in the evaluation of health outcome measures. The techniques are known as differential item functioning (DIF) methods and biased items are said to exhibit DIF.

DIF items have been identified in health outcome measures with respect to gender, age, race, ethnicity, socio-economic status, language, nationality and health care setting [7]. For example, several items from cognitive screening measures were shown to be poor items for those with low education levels [8]. Use of these items may exaggerate the problems of more deprived individuals.

If DIF items are found in measures in development then these items could be re-written or the item could be removed and an alternative DIF-free item with similar item properties could be substituted. If DIF items are found in an existing measure then it may be preferable to select an alternative measure (with no DIF). If data has already been collected then DIF items could be removed and analyses repeated without the DIF items or analyses adjusted to take account of the DIF items.

Importantly, previous studies have shown that cultural differences impact on the validity of items within measures with different DIF items being identified in different countries (e.g. [9,10]). Hence there is a need to explore DIF within UK populations. Furthermore, it has been shown that items work in different ways for different clinical conditions (e.g. [11,12]) including between arthritic conditions e.g. between psoriatic arthritis and rheumatoid arthritis [13].

The WOMAC has alternative formats, it can be administered using visual analogue scale responses or, more commonly, with a 5 point Likert response format. For the Likert-format version, DIF has only been explored in Dutch, Canadian and German patients and in each case DIF items have been identified. DIF items were found for cross-cultural comparisons in the Dutch WOMAC for people with hip OA [14]. Items with DIF were also found with respect to clinical condition and gender in a Canadian community and hip OA sample [15] and with respect to clinical condition in a German population of femoro-acetabular impingement and hip OA [16]. In the UK, the VAS format version has shown DIF items in an osteoarthritis sample with respect to joint (hip v knee) [17].

Thus investigation of the commonly used WOMAC has been very limited for UK populations and the possible biasing effects of social deprivation, psychological state, body mass index or number of affected joints have not been examined.

Hence, the aim of this paper was to examine DIF in the Likert-scaled WOMAC for the first time in a UK osteoarthritis population with respect to demographic, social, clinical and psychological factors. Additionally, the study included factors that had not been explored in any of the other WOMAC DIF studies.

## Methods

### Design

Statistical techniques were applied to an existing data set to explore DIF in items of the WOMAC with respect to demographic, social, psychological and clinical factors.

### Participants

The sample comprised a community sample of 763 people who had been diagnosed with osteoarthritis from 1359 people with hip and/or knee symptoms who completed a follow-up assessment (2002–2003) of health outcome measures as part of the Somerset and Avon Survey of Health Survey (SASH, [18,19]).

SASH is a large scale survey of the population aged 35+. The age–sex stratified survey of 28080 people registered with 40 general practices in Avon and Somerset yielded 2703 people reporting hip and/or knee symptoms at baseline (1994–1995). Osteoarthritis was diagnosed by a clinician assessing X-rays using the Kellgren-Lawrence classification [20].

A written informed consent was obtained from all patients. Ethics approval was obtained from the South West Research Ethics Committee (MREC/01/6/51) and the study was conducted in accordance with the Helsinki Declaration.

### Measures

#### Outcome measures

##### WOMAC

The WOMAC has 24 items with three subscales: pain (5 items), stiffness (2 items) and physical functioning (17 items). Each item was scored 0–4 with a high score indicating a worse outcome. Subscale totals and an overall score were computed. In this study, the pain and stiffness items were asked about hips and knees separately. As the stiffness subscale only had two items, for some analyses it was necessary to combine these 2 items with the pain subscale to form a seven item impairment scale (see Additional file 1: WOMAC items).

##### Subgrouping factors

Median splits were used where appropriate as required for these methods.

The demographic factors explored were gender and age group (median = 70.88), social deprivation was measured by Townsend Index (median = −1.47), social class (1,2,3i v 3ii,4,5) and employment status (paid work v not paid work). Mood was measured using a single item on the EuroQol (no anxiety/depression v moderate or extreme). The clinical factors were Body Mass Index (BMI, normal/overweight v obese i.e., <30,>30), the number of affected OA joints (1or 2 v 3 or 4) and type of OA (hip v knee).

### Statistical analysis

#### General

As the pain and stiffness subscales were measured separately for hip pain/stiffness and knee pain/stiffness only those with diagnosed OA of that joint were included in these analyses.

#### Demographics

T-tests were carried out to explore mean differences on each subgrouping factor on the WOMAC subscales and to explore the relationships between age and the other subgrouping variables.

#### Testing assumptions: unidimensionality

Ordinal factor analysis was carried out to explore unidimensionality. We used the FACTOR computer program [21] using principal component analysis with polychoric correlations. Unidimensionality was supported if there were large difference in eigenvalues between factor 1 to 2 and small difference in eigenvalues between 2 and 3 [22,23]. The number of factors was also evaluated using the MAP procedure proposed by Velicer (1976), [24] which examines the matrix of partial correlations.

### DIF testing

Ordinal Logistic Regression (OLR) was used to explore DIF. In DIF analyses it is crucial to control for the underlying attribute that the item is supposed to be measuring since different groups may have different ability levels (i.e. it is necessary to ‘match’ on ability levels). The total score on the relevant subscale was used as the matching variable.

DIF analysis was carried out by testing the effect of the grouping variable and the interaction term (matching variable by group) once the matching variable has already been added into the model [25,26]. A macro was written in SPSS to facilitate the DIF analysis.

Specifically, the following steps for OLR was carried out for each item.

a) Ordinal logistic regression model:

i) General procedure for DIF testing:

Three OLR models were calculated for each item:

1) Model1: The total score (matching variable) was entered as a predictor variable.

2) Model2: The grouping variable was added into Model1 as a second predictor variable.

3) Model3: The interaction (i.e. group by total) was added into Model2 as the third predictor variable.

The difference in Chi-square between the Model1 and Model3 was tested for the significance as a Chi-Square test with 2 degrees of freedom [25]. If significant, this indicated DIF. The difference in Chi-square between Model2 and Model1 gave a test of uniform DIF (same DIF effect over the construct) and the difference in Chi-square between Model3 and Model2 gave a test of non-uniform DIF (uneven DIF effect over the construct) [25].

Significance testing and item level effect sizes: Different criteria have been suggested to classify items as exhibiting DIF and there appears to be no clear consensus on the best approach. The two most widely used are those proposed by Swaminathan and Rogers (SR) [26] and Zumbo [25]. SR uses a criteria of p < 0.05 for the difference in Chi-square between Model3 and Model1, whereas Zumbo uses p < =0.01. For an item to be classified by Zumbo criteria as having DIF then the effect size must also be significant. This was quantified using effect sizes from Nagelkerke’s R square where the difference in R-squares (Model3/Model1) must be at least 0.035 [27].

However, it has been suggested that Zumbo’s criteria may result in very few items being classified as having DIF, whereas the opposite may be the case for SR i.e. too many items classified [28]. Bonferroni corrections based on test length have been suggested, to minimise Type 1 error due to the multiple testing [29,30]. Hence, here we also applied a Bonferroni correction to the SR method in order to balance not classifying enough items with DIF with classifying too many items with DIF. The three methods are all subsets of each other, i.e. SR would result in the maximum number of DIF items being identified, Zumbo would result in the minimum number of DIF items and applying a Bonferroni correction to SR (SRbon), would result in a number of DIF items between Zumbo and SR. In this study we used all three criteria.

For uniform DIF the odds ratios were calculated to examine the direction of the bias.

ii) Assumption testing: Proportional Odds:

An assumption of ordinal logistic regression is that the parameters coefficients are equivalent across the levels of the dependent variable (i.e. proportional odds). If for any model the assumption of proportional odds was violated then k-1 dichotomous variables were created for that item where k is the number of response categories.

b) Purification:

If DIF items were found then they were removed from the matching variable and all the analyses for the items in that measure re-run. As standard, the item with DIF was included in total for that item as this has been shown to reduce bias [31]. Purification was an iterative process so the analyses may be re-run a number of times until no changes in identified DIF items were seen on two consecutive analyses.

c) Effects of covariates

Where DIF items were identified, the analyses were repeated (steps a-b above) with age additionally entered as a covariate in the logistic regressions to explore if apparent DIF effects in other subgroups were confounded by age.

d) Examination of the impact of DIF at the measure and subscale level.

Modified measures were constructed with DIF items removed and compared to the original measure or subscale. The effect of DIF on group differences was explored using t-tests to see if different conclusions would result if a DIF-free measure was used. Also the difference in significance between the tests was explored by repeated measures ANOVA and exploring the interaction between total and factor i.e. was the effect size reduced by a significant amount depending on the total used. All totals were recalculated as averages due to the different number of items in each total. The impact of the DIF items was based on the results using the SRbon criteria.

e) The validity and reliability of DIF-free measure.

The validity and reliability of DIF-free measures was explored by carrying out standard psychometric tests. Construct validity was explored by examining the relationship of the DIF-free measures with other subscales and measures from other relevant constructs. Internal consistency reliability was explored using Cronbach’s alpha which was calculated for the original measure and for the DIF-free measure.

f) Power:

Based on Crane’s (2006) [29] suggestion for number of participants in each subgroup, we required at least 80 participants per subgroup (based on the maximum of 6 response categories). All subgroups had more participants than the minimum required.

## Results

### Demographics

The participants demographic details are presented in Table 1.

**Table 1:** Participant characteristic table

Gender (male)	43.3%
Age (mean years(s.d.))	69.56 (9.84)
Marital status (married)	72%
Ethnicity (white)	99.1%
Paid employment (yes)	33.7%
Social Class	
I	5.7%
II	33.8%
IIINM	19.0%
III M	23.4%
IV	14.7%
V	3.4%
Townsend quintiles (lower = most affluent)	
20%	−2.76
40%	−1.89
60%	−0.80
80%	1.46
Mood (mod or severe/none)	30%/70%
BMI (mean(s.d.))	29.21 (5.30)
Affected joints	
Hip OA (n)	487
Knee OA (n)	612
Both Hip and Knee OA (n)	336
Hip OA only (n)	151
Knee OA only (n)	276
No of affected joints (mean(s.d.))	2.64 (1.01)
WOMAC physical (mean(s.d.))	20.03 (14.51)
WOMAC pain for Hip OA (mean(s.d.))	3.70(4.16)
WOMAC pain for Knee OA (mean(s.d.))	5.83 (4.34)
WOMAC stiffness Hip OA (mean(s.d.))	1.70 (1.82)
WOMAC stiffness Knee OA (mean(s.d.))	2.53 (1.92)

Being older was associated with being female (t(706) = −2.89 p = 0.004), being in lower social deprivation group (Townsend scores)(t(208.7) = −2.12 p = 0.03), having better mood (t(327.9) = 1.73 p = 0.08) more affected joints (t(543) = −4.43 p < 0.0005), having knee only OA (compared to hip only OA) (t(208.7) = −2.12 p = 0.03) and not working (t(706) = −20.0 p < 0.0005).

Significant mean differences on WOMAC physical function were found with worse physical functioning for those in the lower social class group, the more deprived Townsend group, those not working, in low mood group and being obese, similar differences to those found in other OA samples. Greater knee pain was found associated with the lower social class group, those more deprived, not working, in the low mood group and being obese. More hip pain was found for those younger, in the lower social group, in the low mood group, obese and with fewer affected joints. Greater knee stiffness was associated with the lower social group, not being in paid work, having lower mood and being obese. Greater hip stiffness was associated with being younger, obese, having lower mood and fewer affected joints (see Table 2).

**Table 2:** T-tests WOMAC subscales by subgrouping factors

	**Significance of t-test for each WOMAC subgroup factor and means for each subgroup**				
	**Physical**	**Pain**		**Stiffness**	
**Subgroup factor**		**Knee**	**Hip**	**Knee**	**Hip**
Age	0.07	0.85	**0.002**	0.20	**0.03**
Younger (<=70.88)	18.80	5.72	**4.69**	2.39	** 2.02**
Older (>70.88)	20.88	5.79	**3.44**	2.59	** 1.63**
Gender	0.14	0.12	0.08	0.35	0.12
Male	19.11	5.49	3.72	2.44	1.68
Female	20.76	6.08	4.42	2.60	1.96
Social Class	**<0.0005**	**<0.0005**	**0.04**	**0.006**	0.17
Higher	** 17.86**	**5.23**	**3.72**	**2.35**	1.71
Lower	** 23.04**	**6.73**	**4.59**	**2.80**	1.96
Townsend	**0.001**	**0.002**	0.31	0.13	0.43
Less Deprived	** 18.14**	**5.26**	3.88	2.41	1.76
More Deprived	** 21.97**	**6.42**	4.29	2.66	1.90
Employed	**<0.0005**	**0.007**	0.22	**0.004**	0.41
Paid work	** 15.46**	**5.10**	3.73	**2.19**	1.72
Not paid work	** 22.33**	**6.17**	4.25	**2.69**	1.88
Mood	**<0.0005**	**0.001**	**<0.0005**	**0.003**	**<0.0005**
No anxiety	** 17.76**	**5.44**	**3.51**	**2.38**	**1.55**
Moderate/Extreme	** 25.78**	**6.81**	**5.63**	**2.90**	**2.52**
BMI	**<0.0005**	**<0.0005**	**<0.0005**	**<0.0005**	**0.003**
<30	** 16.26**	**4.96**	**3.45**	**2.18**	**1.61**
>30	**25.65**	**7.21**	**5.12**	**3.04**	**2.16**
Number of joints	0.17	0.78	**0.005**	0.67	**0.001**
1 or 2	18.03	5.64	**5.04**	2.40	**2.34**
3 or 4	19.76	5.76	**3.67**	2.47	**1.62**
Hip only v Knee only	0.77	n/a	n/a	n/a	n/a
Hip	18.18	n/a	n/a	n/a	n/a
Knee	18.69	n/a	n/a	n/a	n/a

Key: Bold, significant t-test; a higher mean reflects a worse WOMAC score.

### Testing assumptions: unidimensionality

The ordinal factor analysis supported the unidimensionality for all subscales of the WOMAC with large difference in eigenvalues between factor 1 to 2 and small difference in eigenvalues between 2 and 3. Only one dimension was also suggested from the MAP procedure. When all the items were combined there was less evidence of unidimensionality with the first factor explaining less variance than the subscales and for hip OA two factors were identified from the MAP analysis. Therefore only the subscales of the WOMAC were explored for DIF (see Table 3).

**Table 3:** Ordinal factor analysis

**WOMAC subscale**	**Eigenvalues (%variance)**			**MAP: number of dimensions**	**N**
	**Factor 1**	**Factor 2**	**Factor 3**		
Physical	12.11 (71.3)	1.27 (7.4)	0.78 (4.6)	1	675
Pain-knee	4.05 (81.1)	0.44 (8.9)	0.28 (5.9)	1	557
Pain-hip	4.39 (87.7)	0.27 (5.4)	0.22 (4.4)	1	449
Impair (stiff + pain)-knee	5.40 (77.2)	0.51 (7.3)	0.42 (6.0)	1	553
Impair (stiff + pain)-hip	5.86 (83.7)	0.44 (6.2)	0.30 (4.3)	1	437
All items-knee	15.96 (66.4)	1.82 (7.6)	1.13 (4.7)	1	517
All items-hip	14.77 (61.5)	3.01 (12.5)	1.08 (4.5)	2	403

Impair, impairment.

### Testing for DIF

#### Physical functioning subscale

##### DIF items

for the WOMAC physical functioning subscale, using the Zumbo criteria only one item was identified for DIF by age and gender, using the SR criteria, 14 of the 17 items were identified as having DIF across the grouping factors, and using the SRbon criteria 5 DIF items were identified (see Table 4).

**Table 4:** DIF testing of WOMAC physical functioning items

	**DIF items using each criteria**			**Type of DIF (SRbon)**	**Impact (p value)**	
**Sub-groups**	**Zumbo**	**SR**	**SRbon**		**t-tests: DIF free items**^**a**^	**Effect size change**^**b**^
Age	**13**	**2,9,**11**,12,13,14**	**2,13,**14	Uniform (+)	0.20	<0.0005
Gender	**13**	**6,**8,9**,12,13 (2,16)**	6,**13**	Uniform(+)	0.32	<0.0005
Social Class	none	**7,16**	none			
Townsend	none	**8,17**	none			
Employed	none	**2,10,12,13,14**	2**,12,14**	12 NU/U; 14 U(−)	<0.0005	0.05
Mood	none	none	none			
BMI	none	**2,3,13,**16,17 **(9,1)**	**2**	U(+)	<0.0005	<0.0005
No joints	none	**13 (6)**	none			
Hip only v Knee only	none	**1,2,11 (7)**	**1**	U(+)	0.88	0.02

Key: Normal text: unpurified DIF items. **Bold**: final purified DIF items. Items in brackets: items that were added during purification. *U* uniform DIF, *NU* non-uniform DIF, + positive odds ratio, - negative odds ratio. ^a^ p-value for t-tests comparing subgroups using DIF free items. ^b^ p-value of ANOVA interaction term to test change in effect size (i.e. the difference between p-values for sub-group comparison using all items v DIF free items).

##### Interpreting DIF effect based on the purified SRbon results

No DIF items were found for the grouping factors of Social group, Townsend Index, Mood or number of affected joints. For item 13 ‘Getting in/out bath’, uniform DIF was identified with women and older people being more likely to respond as having more limitations than men although they had the same actual level of physical functioning. For item 2 ‘Ascending stairs’, older and obese people reported more limitations than they would have with DIF-free items. People not in work tended to score as having less limitations than people in paid work for item 14 ‘sitting’ although they had the same actual level of physical functioning. For item 1 ‘descending stairs’, people with knee OA reported more problems than their ‘true’ level compared to those with hip OA. There was a non-uniform effect for item 12 ‘lying in bed’ by employment (i.e. the effect varied at different levels on the underlying physical functioning construct). At poor overall physical function the responses only slightly differed by employment group, however, in general, at other levels of physical functioning, people not working responded as having more difficulties that people working with the same actual level of physical functioning. However, with age group added into the logistic models as a covariate, while the gender and BMI effects remained, the other significant DIF items were now non-significant (i.e. the 2 DIF items identified for employment status and 1 DIF item for hip v knee OA).

##### Impact: using final purified SRbon

testing for group differences using original and DIF-free measures.

There was a trend (p = 0.07) for differences in physical function by age group (see Table 2). However, this was not significant when the SRbon corrected total was used (p = 0.2). (see Table 4). Across the other grouping factors removal of the DIF items had no impact on conclusions.

Testing for significant differences between original and DIF free measures by group: Using repeated measures ANOVA, significant reduced effects were found using the corrected totals compared to the original totals for all the subgrouping factors.

#### bi) Pain knee subscale

For the pain subscale for those with knee OA, no DIF items were identified using the Zumbo criteria but four of the 5 items did show DIF using the SR criteria for one or more factor (except for social class). No DIF items were identified using SRbon and so no impact analyses were carried out (see Table 5).

**Table 5:** DIF testing WOMAC pain items for participants with Knee OA

	**Age**	**Gender**	**Social class**	**Townsend**	**Employed**	**Mood**	**BMI**	**N**^**o**^**joints**
SR DIF	**5,3**	3,**5**	none	**3**	**3**	**1**	**2**	**3**

Key: Normal text: unpurified DIF items; **Bold**: final purified DIF items.

#### bii) Pain hip subscale

For the pain subscale for those with hip OA, no items were identified for DIF using the Zumbo criteria. Further, DIF was not found for the sub-groups; gender, Townsend deprivation index, mood or number of joints, when assessed by any of three criteria. Three DIF items were identified using SR criteria and 2 items using SRbon (items 2 and 4, see Table 6), however item 4 no longer showed DIF for BMI with age as covariate.

**Table 6:** DIF testing WOMAC pain items for participants with Hip OA

**Sub-group**	**DIF items using each criteria**		**Type of DIF (SRbon)**	**Impact (p value)**	
	**SR**	**SRbon**		**t-tests**	**Effect size change**
Age	**2,**4	**2**	U(+)	0.007	<0.0005
Social Class	3 (5)				
Employed	**2,3,4,**5	2,3			
BMI	**4**	**4**	U and NU	0.0005	0.15

Key: Normal text: unpurified DIF items. **Bold**: final purified DIF items. Items in brackets: items that were added during purification. *U* uniform DIF, *NU* non-uniform DIF, + positive odds ratio, -negative odds ratio. ^a^, p-value for t-tests comparing subgroups using DIF free items. ^b^, p-value of ANOVA interaction term to test the change in effect size (i.e. the difference between p-values for sub-group comparison using all items v DIF free items).

##### Interpreting DIF item effect based on SRbon results

for item 2 ‘Pain going up/down stairs’, uniform DIF was identified with older people reporting more problems than their actual level of functioning would suggest. Non-uniform DIF was identified for item 4 ‘Pain sitting or lying’ by BMI group (see Table 6).

##### Impact: using final purified HIP

There were no differences in conclusions at the scale level using the original or DIF free subscale, however there was a significant reduction in the effect of age on hip pain but there was not a significant difference for the BMI (see Table 6).

#### c.) Stiffness subscale

As the stiffness subscale only contains 2 items, in order to reduce measurement error, these two items were combined with the pain items to form the total score as this was shown as being unidimensional (see Table 3).

For people with knee OA, no items were identified as having DIF. For hip OA, one item (item 2) was identified as having DIF but only when using the SR criteria. As no DIF items were identified using SRbon no impact analyses were carried out.

### The validity and reliability of DIF-free measures

The removal of the 2 DIF items from the physical subscale and the 1 DIF item from pain hip subscale appeared to have only a very small reduction in Cronbach’s alpha (Cronbach’s all = 0.964; without 2 DIF items 0.963). The strength of correlations with the SF-36 physical functioning subscale were only slightly reduced (not shown).

The removal of the 1 DIF item from the pain subscale for those with hip OA also only a very small reduction in Cronbach’s alpha (Cronbach’s all = 0.94; without 1 DIF item =0.93). The strength of correlations with the SF-36 pain subscale were only slightly reduced (not shown).

## Discussion

Overall, the WOMAC performed well with only a small number of DIF items. Five DIF items in the physical functioning subscale were initially identified and two DIF items in the pain subscale for people with hip OA. After adjusting for age, two items were identified for the physical functioning subscale as having DIF with age identified as the DIF factor for 2 items, gender for 1 item and BMI for 1 item. For the item ‘getting in/out bath’ older people and women were more likely to respond as having more limitations than their level of limitation on other items would suggest. The item ‘ascending stairs’ had DIF with those older and obese reporting more limitations than they would have with an unbiased item. For the pain subscale, for people with hip OA, only one item remained after adjusting for age, with older people reporting more pain going up/down stairs than their expected level.

However, previous studies did not identify DIF items for these factors for these subscales [15-17]. All of the previous studies, except one, examined DIF in non-UK patients and this may suggest that the difference is due to lack of cross cultural equivalence. There are many possible explanations for this. It is possible that in the UK older people and women may have more baths than showers than in other countries, or people in the UK may use stairs more as they are more likely to live in houses or to live in flats without a lift, or even structural differences such as the UK having steeper stairs or deeper baths than in other countries. Hence, for older and obese people in the UK the impact of the stairs item may be more pronounced. The study based in the UK that did not identify DIF by age was carried out on the VAS version of WOMAC [17]. Hence it is possible that the method of administration may explain the difference in results.

However there are other explanations that should be considered in comparing this DIF study with others that have been carried out. Differences between our results and previous studies may be due to different analysis methods. All the other studies have used Rasch analysis to explore DIF in the WOMAC. However, the item ‘getting in/out bath’ that we identified as displaying DIF has also been shown not to fit with the Rasch model [15,32] although it was not identified as exhibiting DIF when explored by gender or hip v knee.

At the measure level, different conclusions may have been made if the DIF-free measure was used when exploring the physical functioning subscale by age. In its original form there was a trend of a difference in level of physical function between older and younger people (p = 0.07). However, using the DIF-free measure there was no longer a significant difference between the older and younger people with the p value reducing to 0.20. The impact of the DIF items on BMI for physical functioning subscale and for age for the hip pain subscale appear less likely to change conclusions as although use of the DIF free items did significantly reduce the level of significance, the actual differences were still highly significant.

Therefore it appears that for the physical functioning subscale it would be advisable to analyse data taking into account DIF items when weight, gender or especially age effects, are the focus of interest. Similarly for the pain subscale in people with hip OA it would be worthwhile to analyse data taking into account the possible impact of the DIF items when age comparison are of primary interest. In the study we took the approach of removing the DIF items. However removing items may affect content validity of the measure and comparability with other studies. Using more complex Item Response Theory-based analyses, DIF items do not need to be removed as adjusted scores can be calculated for each subgroup. If the measure is in development, an alternative to deleting the DIF items, may be to substitute similar but DIF-free items either by re-writing, choosing a item with similar item properties or the source of the DIF could be probed by cognitive interviewing or by reviewing the item by groups of experts to detect source of DIF.

The study has some limitations. Different criteria exist for classifying a DIF item using OLR, we used the SR method with a Bonferroni correction. Only the item ‘in/out bath’ by age and gender was identified using the most stringent Zumbo criteria, whereas many DIF items were identified using the SR method and hence it is possible that the impact at scale level would be greater if the purification was based on the SR criteria. However, this was not carried out due to the concern that too many items would be removed and this may also intrinsically change the reliability and validity of the DIF-free measure. We carried out a large number of statistical tests and although we applied a Bonferroni correction it is possible that some findings were due to chance and thus replication would be desirable.

In this study we used OLR to explore DIF due to the accessibility, flexibility and practicality of this method. However, another approach to DIF detection is to use the more complex item response theory (IRT) approach. There is still much debate over the advantages and disadvantages over different methodological approach to DIF [33-35]. IRT does have advantages, in particular the use of the latent variable as the matching variable rather the use of sum scores in OLR. However, IRT is a complex statistical method requiring the use of specialist software and yet produces similar results to OLR [33,34]. Additionally, IRT requires good model fit as poor model fit can contribute to false DIF detection and yet the methods for assessing model fit are not fully established [35]. However, it is possible that we may have got different results if a different DIF method was used. It is also possible that by using different significance criteria for the OLR method we may have reached different conclusions. The study has other limitations. The sample was a community sample and thus had relatively mild OA compared to, say, an arthroplasty sample. However, some of the previous non-UK DIF studies also included a community based OA sample [15], and people with hip OA on their first consultation [16], hence this does allow for our results to be compared with the these studies without differences being attributable to differences in severity levels. However other studies did have arthroplasty participants and so differences between our results and these studies may have been due to the severity of OA in the samples [14,15,17]. The means of WOMAC scores were reported for patients waiting for arthroplasty with the mean for WOMAC physical being 58 (compared to 37 in our sample) and for WOMAC pain, the mean score was 16 compared to 9 in our sample [14]. Additionally, we used median splits and other splits may have produced different results. We also suggest that differences between our study and previous studies may be due to the WOMAC being explored in the UK but it could be that it was due to the specific location including local health service provision. Finally, the diagnosis of OA was based on the health survey followed by x-ray. The patients were not reviewed medically to ascertain that the OA was not coincidental and the hip knee pain did not have another cause and x-rays were not available for all participants and this may have introduced bias.

## Conclusions

Overall the WOMAC performed well with only a small number of DIF items identified across the nine grouping factors. However, DIF items were identified in the WOMAC physical subscale with respect to age, gender and BMI and in the WOMAC pain subscale for people with hip OA with respect to age. The impact of the DIF items rarely had an effect on the conclusions of group comparisons. Nevertheless, it is suggested that when these comparisons are of primary interest, particularly in a UK-based population, analyses should take into account the DIF items. Our findings suggest that there may be social and cultural reasons why items were identified as having DIF in the UK but this will need further exploration.

## Competing interests

The authors declare that they have no competing interests.

## Authors’ contributions

BP participated in the conception and design of the study, the analysis and the drafting and revision of the manuscript. MJ participated in the conception and design of the study and the drafting and revision of the manuscript. DD contributed to the interpretation of the data and revision of the manuscript. All authors read and approved the final manuscript.

## Pre-publication history

The pre-publication history for this paper can be accessed here:

http://www.biomedcentral.com/1471-2474/13/265/prepub

## Supplementary Material

Additional file 1 **The WOMAC items.** Details of the WOMAC items. Click here for file
