# Supplementary material for: Exploring differential item functioning in the Western Ontario and McMaster Universities Osteoarthritis Index (WOMAC)
Source: BMC Musculoskelet Disord. 2012 Dec 29;13:265. doi: 10.1186/1471-2474-13-265 (PMC3582585; doi:10.1186/1471-2474-13-265)
Supplement: Additional file 1 — The WOMAC items. Details of the WOMAC items. [file 1471-2474-13-265-S1.docx]

**The WOMAC items**

| **ITEM NUMBER** | **WOMAC ITEM** |
| --- | --- |
| **Pain subscale** |  |
| 1 | Walking on a flat surface |
| 2 | Going up or down stairs |
| 3 | At night while in bed |
| 4 | Sitting or lying |
| 5 | Standing upright |
|  |  |
| **Stiffness subscale** |  |
| 1 | After first waking in the morning |
| 2 | After sitting, lying or resting later in the day |
|  |  |
| **Physical functioning subscale** |  |
| 1 | Descending stairs |
| 2 | Ascending stairs |
| 3 | Rising from sitting |
| 4 | Standing |
| 5 | Bending to floor |
| 6 | Walking on the flat |
| 7 | Getting in/out of car |
| 8 | Going shopping |
| 9 | Putting on socks/stockings |
| 10 | Rising from bed |
| 11 | Taking off socks/stockings |
| 12 | Lying in bed |
| 13 | Getting in/out of bath |
| 14 | Sitting |
| 15 | Getting on/off toilet |
| 16 | Heavy domestic duties |
| 17 | Light domestic duties |
